# Entertainment Sedentary Time and Subjective Well-Being in College Students: A Moderation Model of Perceived Time Poverty

**DOI:** 10.3390/bs16081408

**Published:** 2026-08-17

**Authors:** Lin-Ming Li, Shi-Yao Wang, Zhi-Lin Jia, Qian Qiu, Feng-Bo Liu, Wei-Wei Sheng, Jing-Jing Ge, Xuan-Yu Zhao, Ruo-Fei Du, Ming Wu

**Affiliations:** 1 School of Kinesiology and Physical Education, Zhengzhou University, Zhengzhou 450001, China; lilinming@gs.zzu.edu.cn (L.-M.L.); wangshiyao@stu.zzu.edu.cn (S.-Y.W.);; 2 China Key Laboratory of Classification, Evaluation and Rehabilitation (Sport) of Intellectual and Development Disability, Zhengzhou University, Zhengzhou 450001, China; 3 School of Physical Education, Xuchang University, Xuchang 461000, China; 4 School of Physical Education, Zhengzhou University of Light Industry, Zhengzhou 450001, China; 5 School of Physical Education, Zhengzhou University of Science and Technology, Zhengzhou 450064, China

**Keywords:** entertainment sedentary time, subjective well-being, perceived time poverty, college students, moderation

## Abstract

This study examined the association between entertainment sedentary time (EST) and subjective well-being (SWB) among Chinese college students and further explored the moderating role of perceived time poverty (PTP). A total of 1582 college students completed questionnaires assessing EST, PTP, SWB, and moderate-to-vigorous physical activity (MVPA). Descriptive statistics, correlation analyses, multiple linear regression analysis, and moderation analysis using Model 1 of the PROCESS macro for SPSS (Version 4.2) were conducted. After adjustment for gender, grade, and MVPA, EST was significantly and negatively associated with SWB, although the association was weak. PTP was also significantly and negatively associated with SWB. The interaction between EST and PTP was statistically significant, indicating that PTP moderated the association between EST and SWB. Specifically, the negative association between EST and SWB was stronger among students with lower levels of PTP and weakened as PTP increased. Johnson–Neyman analysis further identified the ranges of PTP within which the conditional association between EST and SWB was statistically significant. These findings suggest that PTP may function as a contextual boundary condition of the association between EST and SWB and should be considered when interpreting this association.

## 1. Introduction

Subjective well-being (SWB) refers to individuals’ cognitive and affective evaluations of their lives and comprises three core dimensions: life satisfaction, positive affect, and negative affect (Diener, 1984). Higher levels of SWB are generally associated with better mental health, more positive interpersonal relationships, and better social adjustment, and SWB is considered an important psychological indicator of positive development among college students (Diener et al., 2018). The college years involve multiple challenges, including academic demands, changes in interpersonal relationships, and lifestyle adjustments. Therefore, gaining a better understanding of the factors associated with college students’ SWB is of considerable practical importance for supporting their mental health.

With the development of digital media, screen-based entertainment activities have become an important part of college students’ daily leisure, and entertainment sedentary time (EST) has become increasingly common (Carpenter et al., 2021). Existing research has not reached a consistent conclusion regarding the association between EST and SWB. Some studies have found that higher levels of EST are associated with lower levels of SWB (Brar et al., 2026). According to the displacement hypothesis (Neuman, 1988), longer EST may coincide with less time devoted to physical activity, peer interaction, and academic pursuits, all of which are considered closely associated with positive psychological functioning (Passeggia et al., 2023; Sandstrom & Dunn, 2014; Z. Zhang et al., 2021). Other studies have suggested that specific forms of social media use may be associated with higher levels of SWB (C. Zhang et al., 2023).

These inconsistent findings suggest that the association between EST and SWB cannot be fully understood by considering EST alone and may depend on additional factors. Previous research has primarily examined associations between the duration, frequency, and type of entertainment sedentary activities and well-being, while paying comparatively less attention to individuals’ subjective time context (Kaye et al., 2020; Vanden Abeele, 2021). Because the same duration of EST may have different psychological meanings under different subjective time conditions, examining duration alone may be insufficient to explain variations in the association between EST and SWB. Therefore, examining perceived time poverty (PTP) as a potential boundary condition of the association between EST and SWB may help refine existing research models. Accordingly, the present study examined the association between EST and SWB among college students and further tested the moderating role of PTP.

### 1.1. Entertainment Sedentary Time and Subjective Well-Being

EST refers to time spent engaging in entertainment for nonacademic and nonwork purposes while sitting, lying, or reclining in low-energy-expenditure postures (Tremblay et al., 2017). Among Chinese college students, daily smartphone screen time typically ranges from 3 to 7 h/day, and EST is generally reported at relatively high levels (Carpenter et al., 2021; Fang et al., 2023). Given that EST constitutes an important component of college students’ daily time allocation, its association with SWB warrants further investigation.

Self-Determination Theory (SDT) provides a possible theoretical perspective for understanding the association between EST and SWB. SDT proposes that the satisfaction of three basic psychological needs, namely autonomy, competence, and relatedness, is an important source of individual well-being (Ryan & Deci, 2000). Entertainment sedentary activities, such as watching videos, browsing social media, and playing video games, may provide opportunities to satisfy these needs through autonomous content choices, experiences of competence, and online social connection and may therefore be associated with higher levels of SWB. However, longer EST may also coincide with less time devoted to developmentally relevant activities, such as academic work, physical activity, and offline social interaction, and may thus be associated with fewer opportunities for basic psychological need satisfaction and lower levels of SWB (West et al., 2024). In other words, the association between EST and SWB may vary according to how entertainment sedentary activities are used. Empirically, existing research has not reached a consistent conclusion regarding the association between EST and SWB, and much of the available evidence has focused on children and adolescents. On the one hand, because EST may provide immediate relaxation and enjoyment, it may be associated with short-term improvements in emotional states (Chen et al., 2022). On the other hand, large-sample studies and systematic reviews have found that longer screen time is generally associated with lower life satisfaction and psychological well-being (Twenge et al., 2018; Stiglic & Viner, 2019). Using data from the U-Flourish longitudinal survey of college student health, Brar et al. (2026) similarly found, through a longitudinal cohort design and multivariable regression analyses, that longer recreational screen time was associated with lower well-being and greater psychological distress among freshmen. However, these associations are generally weak and may vary according to the type and context of screen-based activities (Orben & Przybylski, 2019). Overall, the available empirical evidence more consistently points to a negative association between EST and SWB. Based on the existing theoretical and empirical evidence, the present study proposed the following hypothesis:

**H1.**  *EST is negatively associated with SWB among college students.*

### 1.2. The Moderating Role of Perceived Time Poverty

Perceived time poverty (PTP) refers to a psychological state in which individuals subjectively perceive that the time available to them is insufficient to complete necessary or desired activities (X. Zheng et al., 2022). It differs from an objective lack of free time by emphasizing individuals’ subjective evaluations of the availability, controllability, and allocation of their time. Previous studies have shown that PTP is associated with greater psychological stress and lower SWB, whereas perceived time affluence is associated with higher well-being and greater satisfaction of psychological needs (Giurge et al., 2020; Kasser & Sheldon, 2009). Diary-based research has also shown that individuals’ subjective perceptions of whether they have sufficient time are more closely associated with daily well-being than the objective amount of time available (Dong & Sun, 2025).

Previous research has primarily examined the direct association between EST and SWB, with comparatively limited attention to the subjective time context in which entertainment activities occur. However, the same amount of EST may occur under different conditions of perceived time availability. Autonomous leisure after completing academic tasks may differ from screen-based entertainment while tasks remain unfinished in terms of opportunity costs, perceived control over time, and evaluations of the activity. Therefore, examining the duration of EST alone may be insufficient to explain the inconsistent associations between EST and SWB. This highlights the need to incorporate PTP into the analytical framework and examine its moderating role in this association.

From the perspective of the psychological meaning of time resources, different levels of PTP may shape how EST is experienced and evaluated. When individuals perceive that they have relatively sufficient time, aimless leisure activities may be more likely to elicit a negative sense of wasting time (Sharif et al., 2021). de Segovia Vicente et al. (2024) found that mindless scrolling through social media feeds without a clear purpose was associated with greater goal conflict and guilt about smartphone use, and these negative experiences were in turn associated with lower daily well-being. Accordingly, under conditions of relatively low PTP, longer EST may be more likely to highlight perceived inefficiency in time use and may therefore be negatively associated with SWB. When individuals perceive their time as constrained, EST may serve a different psychological function. Under sustained task pressure and low perceived control over time, brief digital entertainment may be viewed as one of the few available opportunities for recovery. The recovery theory suggests that temporary psychological detachment from task demands may help alleviate ongoing strain and restore psychological resources (Sonnentag & Fritz, 2007). Related empirical research has also found that individuals use video games to recover from stress and fatigue and that subjective recovery experiences are closely associated with such entertainment use (Reinecke, 2009). Accordingly, under conditions of greater perceived time pressure, EST may be experienced as having some restorative value, and the negative association between EST and SWB may therefore vary.

In summary, individuals may experience and evaluate EST differently across levels of PTP, and the association between EST and SWB may therefore vary accordingly. However, because the two possible explanations described above may coexist and operate in different directions, the specific pattern of moderation cannot be predicted with confidence. Accordingly, the present study proposed the following exploratory hypothesis:

**H2.**  *PTP moderates the association between EST and SWB among college students.*

## 2. Materials and Methods

### 2.1. Participants and Procedures

Convenience sampling was used to recruit students enrolled at universities in Henan Province, China, between March and May 2025. The survey was administered online through Wenjuanxing (www.wjx.cn) and included attention-check items. Completion required approximately 15–25 min. Before participation, the researchers explained the purpose and procedures of the study, emphasized that participation was voluntary, and informed participants that they could withdraw at any time without penalty. During survey completion, participants were instructed to distinguish EST carefully from other forms of sitting time to avoid double counting. Following data collection, all responses were screened for completeness, the plausibility of the reported time-use data, and logical consistency. A total of 1650 questionnaires were returned. Responses were excluded if they met any of the following criteria: (1) an implausibly short or long completion time; (2) missing data on more than one third of the items; (3) invariant or patterned responding, such as straight-line or wave-like response patterns; or (4) failure to pass an attention check. After exclusions, 1582 valid questionnaires remained, corresponding to a valid-response rate of 95.9%. The final sample included 746 men (47.2%) and 836 women (52.8%), indicating a relatively balanced gender distribution. By grade, 570 participants were freshmen (36.0%), 895 were sophomores (56.6%), 88 were juniors (5.6%), and 29 were seniors (1.8%).

The minimum required sample size was estimated using G*Power (Version 3.1.9.7; Faul et al., 2009). An F-test for the increase in R^2^ in linear multiple regression was specified with an effect size of *f*^2^ = 0.03, *α* = 0.05, power (1 − *β*) = 0.95, one tested predictor, and eight total predictors. These parameters yielded a minimum required sample of 436 participants. The final sample (*N* = 1582) substantially exceeded this requirement, indicating adequate statistical power. The study was conducted in accordance with the Declaration of Helsinki and was approved by the Ethics Committee of the School of Physical Education, Zhengzhou University (main campus; approval no. ZZUIRB2024-0010).

### 2.2. Measures

#### 2.2.1. Entertainment Sedentary Time (EST)

EST was assessed using the item on electronic screen-based device use for entertainment from the 24-Hour Movement Behaviors Questionnaire (24HMBQ) for Chinese College Students developed by J. Zheng et al. (2023). The questionnaire has demonstrated good content validity, test–retest reliability, and convergent validity in Chinese college students. It includes three parts: sleep, sedentary behavior, and physical activity. A single item was extracted from the sedentary behavior section as the operational indicator of EST: “During the past week, on average, how much time per day did you spend on electronic screen-based devices for entertainment while sitting or lying?” To distinguish EST from other forms of sedentary behavior, participants were provided with the following instruction: “do not count the time using electronic screen-based devices for study or work, but including watching TV/movies/short videos, video gaming, and using social media, etc.” Accordingly, the EST assessed in the present study primarily included screen-based entertainment activities such as watching television, movies, and short videos, playing video games, and using social media. The item is divided into two parts: weekdays and weekend days. The test–retest reliability was excellent for weekdays (ICC = 0.97, 95% CI: 0.96–0.98) and good for weekends (ICC = 0.75, 95% CI: 0.67–0.80) (J. Zheng et al., 2023).

For the purpose of this study, the average EST (hours/day) was calculated using a weighted formula: (average EST on weekdays × 5 + average EST on weekend days × 2) / 7. This variable was then treated as a continuous measure in the analyses.

#### 2.2.2. Subjective Well-Being (SWB)

SWB was assessed using the measure administered to Chinese college students by X. Zheng et al. (2001), which was derived from the International College Survey developed by Diener et al. (2001). The measure contains 19 items across three subscales: life satisfaction, positive affect, and negative affect. Items are rated on a 7-point Likert-type scale ranging from 1 (not at all true) to 7 (completely true). Following the approach used by Sheldon and Niemiec (2006), an overall SWB score was computed by adding the life-satisfaction and positive-affect scores and subtracting the negative-affect score. Higher scores indicated greater SWB. In the present study, Cronbach’s *α* was 0.883 for the total scale and 0.915, 0.929, and 0.939 for the three subscales, respectively.

#### 2.2.3. Perceived Time Poverty (PTP)

PTP was assessed using the six-item unidimensional Perceived Time Poverty Scale developed by X. Zheng et al. (2022). A sample item is “I often feel like there is no time for personal recreation.” Items are rated on a 7-point Likert-type scale ranging from 1 (strongly disagree) to 7 (strongly agree). Item scores were summed, with higher total scores indicating greater PTP. Cronbach’s *α* was 0.953 in the present study.

#### 2.2.4. Moderate-to-Vigorous Physical Activity (MVPA)

Moderate-to-vigorous physical activity (MVPA) was assessed using the physical activity section of the 24-Hour Movement Behaviors Questionnaire (24HMBQ) for Chinese College Students developed by J. Zheng et al. (2023). This section assesses vigorous-, moderate-, and light-intensity physical activity during the past 7 days across three domains: daily exercise, including workouts and physical education classes, daily transportation, and daily dormitory life. The present study used the six items assessing vigorous- and moderate-intensity physical activity across these three domains. For each item, participants reported the number of days per week on which they performed the corresponding activity and the average duration of the activity in hours and minutes per day on days when the activity was performed. The weekly duration of each activity was first calculated by multiplying the number of days per week by the reported duration per day. The durations of vigorous- and moderate-intensity physical activity across the three domains were then summed to obtain total weekly MVPA. This value was divided by 7 and converted to hours per day to calculate average daily MVPA. Higher values indicated a longer average daily duration of MVPA. MVPA was included as a continuous covariate in the subsequent analyses.

### 2.3. Data Analysis

IBM SPSS Statistics for Windows (Version 27.0) was used for data management and statistical analysis. First, descriptive statistics and correlation analyses were conducted. Means, standard deviations, and Pearson correlation coefficients were calculated for the main variables to provide an initial description of their patterns of association. Multiple linear regression analysis was then used to examine the association between EST and SWB, with gender, grade, and MVPA included as covariates. Grade was treated as a categorical variable, with freshmen as the reference group. Three dummy variables were created for sophomores, juniors, and seniors, with membership in the corresponding grade coded as 1 and all other grades coded as 0. This approach avoided the assumptions of equal intervals and a linear relationship that would be implied by treating grade as a continuous variable.

Moderation analysis was conducted using Model 1 of the PROCESS macro for SPSS (Version 4.2; Hayes, 2018) to examine whether the association between EST and SWB varied across levels of PTP. EST and PTP were mean-centered before their product term was constructed. When the interaction term was statistically significant, simple slope analyses were conducted at low, mean, and high levels of PTP, defined as the mean minus one standard deviation, the mean, and the mean plus one standard deviation, respectively. These analyses estimated the association between EST and SWB at different levels of PTP, and an interaction plot was produced. In addition, the Johnson–Neyman technique was used to identify the ranges of PTP within which the conditional association between EST and SWB was statistically significant.

## 3. Results

### 3.1. Preliminary Assessment of Common Method Variance

Because all study variables were assessed using self-report measures, the data may have been affected by common method variance. Harman’s single-factor test was therefore conducted as a preliminary diagnostic. All relevant questionnaire items were entered into an unrotated exploratory factor analysis. Seven factors with eigenvalues greater than 1 were extracted, and the first factor accounted for 26.86% of the total variance, which was below the commonly used 40% reference criterion (Zhou & Long, 2004). Thus, the analysis did not reveal clear evidence of substantial common method variance.

### 3.2. Descriptive Statistics and Correlation Analyses

Descriptive statistics and correlations among the study variables are presented in Table 1.

College students reported an average of 4.75 h/day of EST and 1.73 h/day of MVPA. EST was negatively correlated with SWB (*r* = −0.107, *p* < 0.001) and MVPA (*r* = −0.076, *p* = 0.002) and positively correlated with PTP (*r* = 0.087, *p* < 0.001). PTP was negatively correlated with SWB (*r* = −0.272, *p* < 0.001) but was not significantly correlated with MVPA. MVPA was positively correlated with SWB (*r* = 0.056, *p* = 0.025).

### 3.3. Regression Analysis of EST and SWB

To examine the association between EST and SWB, collinearity diagnostics were first conducted for all predictor variables. The VIF values ranged from 1.026 to 1.133, all of which were below the threshold of 5, indicating that multicollinearity was not a serious concern. A multiple linear regression analysis was then performed after adjustment for gender, grade, and MVPA. The results are presented in Table 2.

After adjustment for gender, grade, and MVPA, EST was significantly and negatively associated with SWB (*B* = −0.642, *β* = −0.108, 95% CI [−0.936, −0.348], *p* < 0.001). In terms of the original measurement units, each additional hour per day of EST was associated with a 0.642-point lower estimated SWB score. The overall regression model was statistically significant, *R*^2^ = 0.021, adjusted *R*^2^ = 0.017, *F*(6, 1575) = 5.688, *p* < 0.001, and accounted for 2.1% of the variance in SWB. Compared with freshmen, sophomores had significantly higher SWB (*B* = 2.529, *β* = 0.088, 95% CI [1.037, 4.022], *p* < 0.001), whereas the differences between juniors and freshmen and between seniors and freshmen were not statistically significant.

### 3.4. Moderation Analysis

Model 1 of the PROCESS macro for SPSS (Version 4.2; Hayes, 2018) was used to examine whether PTP moderated the association between EST and SWB. After adjustment for gender, grade, and MVPA, mean-centered EST and PTP and their interaction term were entered into the model.

The overall model was statistically significant, *R*^2^ = 0.101, *F*(8, 1573) = 22.078, *p* < 0.001, accounting for 10.1% of the variance in SWB. When PTP was at its mean level, EST was significantly and negatively associated with SWB (*B* = −0.558, *β* = −0.094, 95% CI [−0.842, −0.274], *p* < 0.001). When EST was at its mean level, PTP was also significantly and negatively associated with SWB (*B* = −0.490, *β* = −0.258, 95% CI [−0.580, −0.401], *p* < 0.001). The interaction between EST and PTP was statistically significant (*B* = 0.085, *β* = 0.108, 95% CI [0.048, 0.122], *p* < 0.001) and accounted for an additional 1.2% of the variance in SWB (Δ*R*^2^ = 0.012, *f*^2^ = 0.013). The positive interaction coefficient indicated that the negative association between EST and SWB became weaker as PTP increased. The results are presented in Table 3.

**Figure 1:**
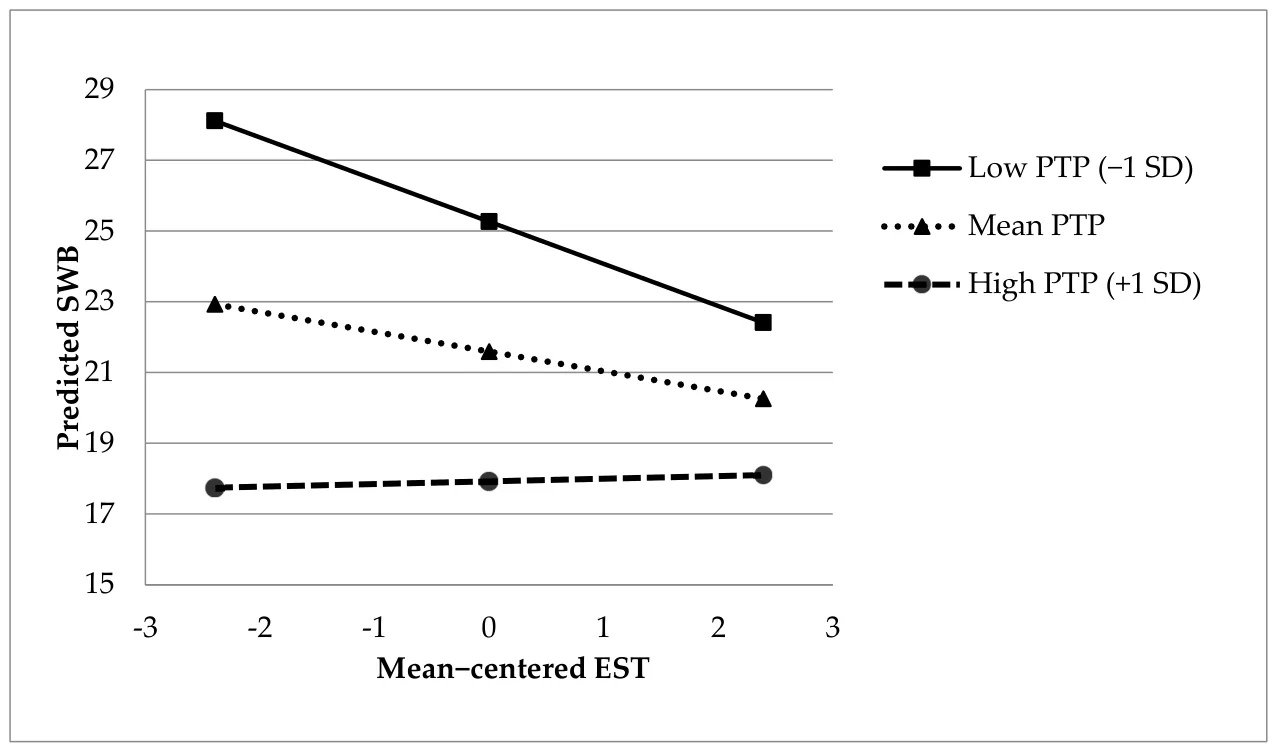
Conditional association between EST and SWB at different levels of PTP. Note: Low, mean, and high levels of PTP correspond to *M* − 1 *SD*, *M*, and *M* + 1 *SD*, respectively. Predicted SWB values were estimated from the moderation model after adjustment for gender, grade, and MVPA.

To further probe the significant interaction, simple slope analyses were conducted at low (*M* − 1 *SD*), mean (*M*), and high (*M* + 1 *SD*) levels of PTP. The detailed results are presented in Table 4, and the interaction pattern is illustrated in Figure 1.

The results showed that, at a low level of PTP, EST was significantly and negatively associated with SWB (*B* = −1.191, *SE* = 0.210, 95% CI [−1.604, −0.779], *p* < 0.001). At the mean level of PTP, the negative association between EST and SWB was weaker but remained statistically significant (*B* = −0.558, *SE* = 0.145, 95% CI [−0.842, −0.274], *p* < 0.001). At a high level of PTP, EST was not significantly associated with SWB (*B* = 0.075, *SE* = 0.194, 95% CI [−0.305, 0.455], *p* = 0.700).

**Figure 2:**
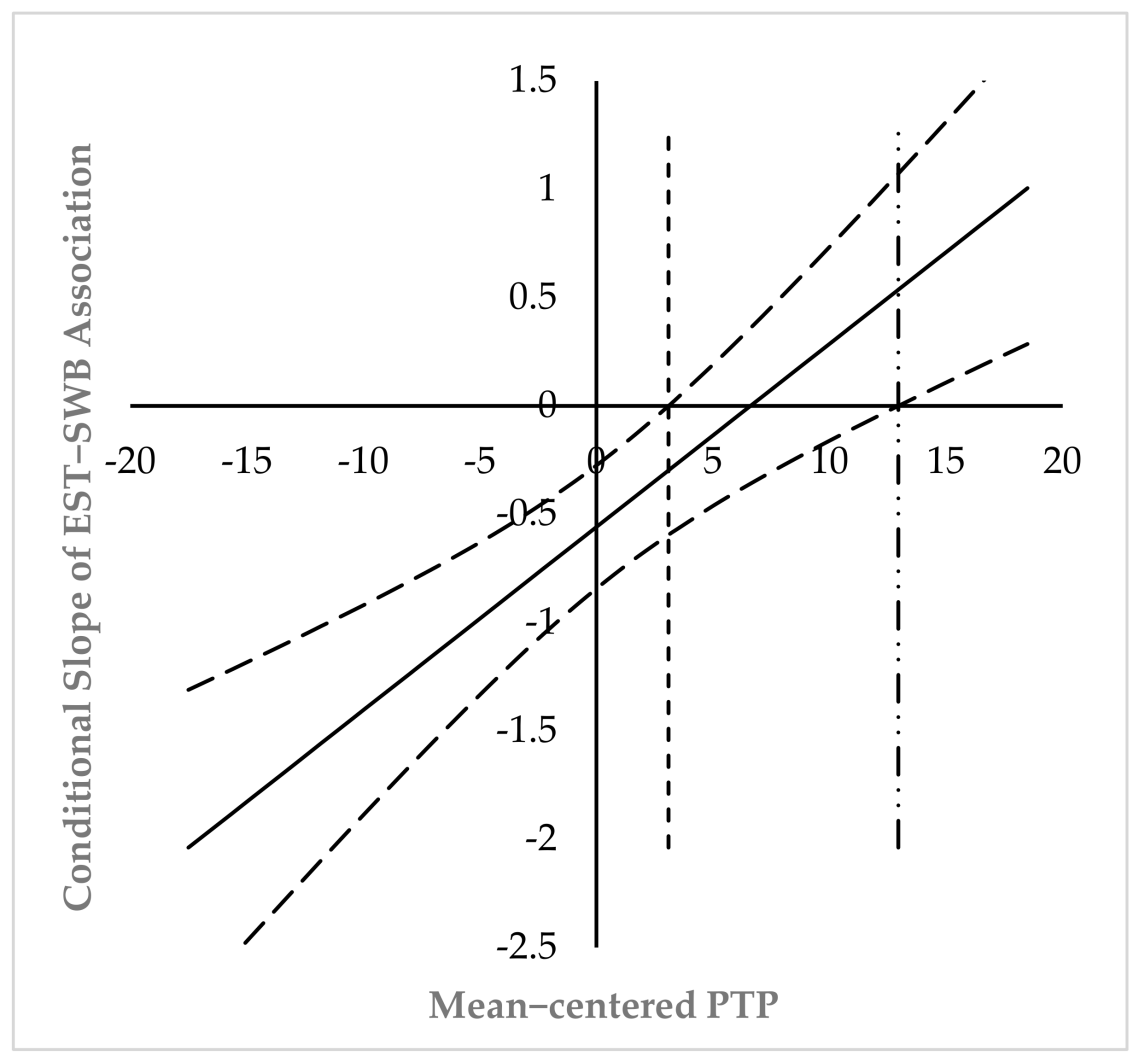
Johnson–Neyman plot of the conditional association between EST and SWB across levels of PTP. Note: The solid line represents the conditional slope of EST in relation to SWB, and the dashed lines represent its 95% confidence interval. The horizontal solid line indicates a conditional slope of zero. The two vertical reference lines indicate the Johnson–Neyman threshold values.

Johnson–Neyman analysis further identified the regions in which the conditional association between EST and SWB was statistically significant (Figure 2). When raw PTP scores were below 26.59 (mean-centered value < 3.08), the conditional association between EST and SWB was significantly negative. When raw PTP scores ranged from 26.59 to 36.46 (mean-centered values from 3.08 to 12.95), the conditional association was not statistically significant. When raw PTP scores were above 36.46 (mean-centered value > 12.95), the association between EST and SWB was significantly positive.

## 4. Discussion

### 4.1. Association Between EST and SWB

The present study found that, after adjustment for gender, grade, and MVPA, EST was significantly and negatively associated with SWB among college students, supporting H1. The standardized regression coefficient for EST was small (*β* = −0.108), indicating a weak association. In terms of the original measurement units, each additional hour per day of EST was associated with a 0.642-point lower estimated SWB score. The overall regression model accounted for 2.1% of the variance in SWB. Taken together, the small standardized regression coefficient and the limited explanatory power of the overall model suggest that EST is more appropriately regarded as a behavioral factor weakly associated with SWB rather than a major determinant of SWB. Given the high prevalence of entertainment screen use among college students, even a small association may still have cumulative value from a population health perspective (Götz et al., 2022; Carey et al., 2023). Its actual public health significance still needs to be confirmed through longitudinal and intervention studies.

In addition, the analysis of grade as a covariate showed that sophomores had significantly higher SWB than freshmen. This difference may be related to stages of adjustment to college: freshmen are still transitioning from high school to college and need to adapt to new learning approaches, living environments, and interpersonal relationships, whereas sophomores may have gradually completed the initial adjustment (Meng et al., 2015). Nevertheless, given the cross-sectional design of the present study and the unequal sample sizes across grade levels, this interpretation should be viewed with caution.

The weak negative association between EST and SWB observed in the present study is broadly consistent with the general pattern reported in previous research. Brar et al. (2026) found that longer recreational screen time generally coincided with lower well-being and greater psychological distress, although some longitudinal associations were substantially attenuated after adjustment for baseline psychological status. de la Rosa et al. (2025) similarly reported that associations between different types of recreational screen activities and subsequent indicators of well-being were generally small and varied across activity types. Although the behavioral and well-being measures used in these studies were not identical to EST and SWB as assessed in the present study, the findings suggest that associations between recreational screen use and well-being are generally modest and may vary according to pre-existing psychological status and the specific type of activity. Orben and Przybylski (2019) likewise found that digital technology use accounted for only a small proportion of the variance in well-being and that its association with well-being may differ across specific activities and contexts. However, previous evidence has not shown a consistently negative pattern. Studies focusing on social and relational forms of use suggest that, under certain circumstances, digital media activities may be associated with higher levels of well-being through self-esteem, online social support, or prosocial behavior (C. Zhang et al., 2023; Hui et al., 2023). Studies using objective behavioral records likewise yielded no single consistent pattern: platform-recorded video game play showed a small positive association with affective well-being, whereas most prospective associations involving device-recorded smartphone or social media use were close to zero (Johannes et al., 2021; Sewall et al., 2022). Separately, an eight-year longitudinal questionnaire study reported largely null within-person associations between social media time and depression or anxiety (Coyne et al., 2020). This heterogeneity may reflect differences in sample characteristics, cultural backgrounds, and measurement methods. Digital entertainment experiences may vary across developmental stages and cultural contexts, and objectively recorded behavior and self-reported duration may not capture exactly the same aspects of use. Given that the present study combined multiple forms of screen-based entertainment into a self-reported measure of total EST and that the sample consisted mainly of lower-year university students from Henan Province, the observed weak negative association is more appropriately interpreted as an overall pattern of association within this specific sample.

The displacement hypothesis provides a possible theoretical perspective for understanding the weak association observed in the present study. This perspective proposes that individuals have a limited amount of time available each day, such that more time devoted to one activity necessarily leaves less time for other activities (Neuman, 1988). Entertainment sedentary activities are not necessarily negative in themselves, but longer periods of engagement may coincide with less time available for sleep, face-to-face interaction, active leisure, or academic activities, all of which are closely associated with well-being and life satisfaction among college students (Kim et al., 2024). However, the present study did not assess which specific activities were displaced by EST. Therefore, time displacement can only be regarded as a possible explanation. The negative coefficient for EST remained statistically significant after adjustment for MVPA, suggesting that the association cannot be fully attributed to differences in the measured level of physical activity. Nevertheless, this finding does not demonstrate that the observed association is independent of other unmeasured behavioral or psychological factors.

SDT further suggests that the psychological meaning of an activity depends not only on its duration but also on whether it satisfies the needs for autonomy, competence, and relatedness (Ryan & Deci, 2000). Entertainment activities that have a clear purpose, promote social connection, or provide experiences of competence may have different psychological implications from use characterized by passivity, loss of control, or subsequent regret. de Segovia Vicente et al. (2024) found that aimless scrolling through social media feeds was associated with greater goal conflict and guilt about smartphone use, and that these negative experiences were further associated with lower daily well-being. This evidence suggests that simply measuring the total duration of EST may obscure differences in activity content, motivations for use, and subjective experiences. The present study used a single item covering activities such as television viewing, short-video viewing, video gaming, and social media use. Therefore, it cannot determine which type of activity was most closely associated with the observed negative association or examine whether autonomy, goal conflict, or psychological need satisfaction was involved. The theoretical significance of the present study does not lie in suggesting that EST is generally detrimental to well-being. Rather, it highlights the need for future research to pay greater attention to how entertainment time is used, which activities are displaced, and individuals’ subjective evaluations of their time use.

Taken together, the existing evidence suggests that the association between EST and subjective well-being is generally weak and may vary according to activity type, baseline psychological status, and context of use. Therefore, future intervention studies should not focus solely on total EST duration but should further examine whether purposeless or uncontrolled use, as well as the displacement of sleep, study, physical activity, and offline social interaction by entertainment activities, has greater practical relevance. On this basis, university health promotion programs may use longer EST as a cue to further understand students’ specific patterns of use and overall lifestyles, rather than treating it as a target for behavioral correction based solely on duration. Future intervention studies could also compare whether helping students establish entertainment routines with clear boundaries and purposes has greater practical value than simply reducing EST. It should be emphasized that, given the cross-sectional design of the present study and the limited effect sizes observed, these suggestions represent only intervention directions that require further validation. The present study can demonstrate only a statistical association between EST and subjective well-being; it cannot determine the temporal ordering or causal direction of this association, nor can it rule out the reverse association that students with lower subjective well-being may be more likely to engage in EST. Future research could employ longitudinal or intervention designs, further distinguish among types of entertainment activities, and measure variables such as psychological need satisfaction, motivations for use, and activity displacement to examine the potential mechanisms and boundary conditions.

### 4.2. The Moderating Role of PTP in the Association Between EST and SWB

The present study found that PTP significantly moderated the association between EST and SWB among college students, supporting H2. As PTP increased, the negative association between EST and SWB gradually weakened. At a low level of PTP, EST was significantly and negatively associated with SWB. At the mean level of PTP, the association remained significant but was weaker, whereas at a high level of PTP, it was no longer statistically significant. The interaction term accounted for an additional 1.2% of the variance in SWB, indicating that PTP may constitute a contextual boundary condition of the association between EST and SWB. However, its incremental explanatory power remained limited, and this result should therefore be regarded as exploratory.

It should be noted that the weakening of the negative slope as PTP increased does not imply that PTP played a protective role. PTP itself was significantly and negatively associated with SWB, indicating that greater perceived time constraints were still associated with lower SWB. More precisely, among students who perceived their time as relatively sufficient, differences in EST were more closely associated with differences in SWB. Among students with high levels of PTP, the association between EST and SWB was comparatively weaker. This finding suggests that PTP is a contextual variable worthy of attention when examining the association between EST and SWB. Previous research has primarily focused on the direct association between EST and SWB, but the same amount of EST may occur under different time conditions. Whether individuals perceive that they have sufficient time is related not only to the objective amount of discretionary time available but also to their evaluations of task demands, autonomy over time, and the value of activities (Roxburgh, 2004; Giurge et al., 2020).

When PTP was high, the negative association between EST and SWB was no longer statistically significant, and different explanations may be possible. On the one hand, under conditions of intensive task demands and time pressure, brief screen-based entertainment may be perceived as one of the few available opportunities for rest. Recovery theory may provide a plausible explanation for this finding. Temporary psychological detachment from task demands may help restore psychological resources. The immediate pleasure derived from entertainment activities may be part of this short-term recovery experience, particularly when discretionary time is limited and leisure activities are autonomously chosen. If entertainment activities displace sleep, study, physical activity, or offline social interaction, these immediate benefits may coexist with opportunity costs. The same activity may provide short-term pleasure or recovery while showing a different association with SWB at the level of overall life evaluation. However, because the present study did not directly measure these processes, this explanation requires further examination in future research (Sonnentag & Fritz, 2007). On the other hand, extremely high PTP may itself reflect excessive academic workload, difficulties in time management, or psychological distress, and these factors may be more strongly associated with variation in SWB. Regardless of which explanation is more plausible, both suggest that the negative association between EST and SWB weakens under conditions of high PTP and may no longer be statistically significant.

Johnson–Neyman analysis showed that, when PTP reached a relatively high level, the conditional slope of EST in relation to SWB became positive. This range included only approximately 4.2% of the participants and was located at the extreme tail of the sample distribution. This positive association may reflect instability in estimates at the tail of the distribution or a tendency for individuals experiencing severe time pressure to seek immediate psychological detachment through entertainment activities. This finding should be interpreted with caution and should not be used as a basis for encouraging students to increase EST.

The findings of the present study may provide some practical insights into the relationship between EST and students’ time circumstances. For students who perceive their time as relatively sufficient but report longer EST, future research may further examine the practical value of addressing purposeless use and the displacement of sleep, study, and offline activities. For students with higher levels of PTP, research may also examine whether prioritizing the assessment of academic workload, perceived control over time, and psychological distress is more appropriate than simply reducing their limited leisure time. Assessing EST alongside PTP may inform the design of university health promotion and psychological support measures, although the practical value of this approach requires further evaluation in longitudinal or intervention studies.

### 4.3. Limitations

Using cross-sectional data, the present study provided preliminary evidence of the association between EST and SWB and suggested that PTP may serve as a contextual boundary condition. Several limitations should be acknowledged.

First, the cross-sectional design provided preliminary evidence but could not determine the temporal order or causal direction of the variables, nor could it rule out reverse or bidirectional associations. College students with lower SWB may also spend more time engaging in EST.

Second, EST, PTP, SWB, and MVPA were all assessed using self-report measures and may therefore have been affected by recall bias, social desirability bias, and individual response tendencies. In particular, EST and MVPA required participants to retrospectively estimate the duration of their activities over the past week, and the associated measurement error may have been more pronounced. Notably, the mean MVPA in the present study was 1.73 h/day, which may have been overestimated because of recall bias arising from self-report, a relatively common issue in physical activity research. Although Harman’s single-factor test did not indicate substantial common method variance, it is only a preliminary diagnostic. Because all key variables were collected at the same time point from the same participants using self-report measures, common method variance cannot be ruled out.

Third, EST was operationalized using a single self-report item. Although this item was drawn from the 24HMBQ and showed acceptable test–retest reliability in previous validation research, a single-item measure provides limited construct coverage and does not allow the assessment of internal consistency. In addition, the item combined activities such as television viewing, short-video viewing, video gaming, and social media use into an overall measure of EST, making it impossible to distinguish activity content, purpose of use, mode of interaction, and context of use. Social media use, video gaming, streaming media viewing, and passive browsing may show different patterns of association with SWB.

Fourth, although the study controlled for gender, grade, and MVPA, PTP may also reflect, to some extent, academic stress, task demands, difficulties in time management, or psychological distress. These factors were not measured simultaneously in the present study, which limits the independent interpretation of the moderating role of PTP.

Fifth, the present study used convenience sampling, and all participants were recruited from universities in Henan Province, China, resulting in limited regional and institutional coverage. In addition, the sample consisted primarily of freshmen and sophomores, who together accounted for 92.6% of the participants, whereas juniors and seniors accounted for only 5.6% and 1.8%, respectively. Therefore, the applicability of the present findings to other regions, different types of universities, and juniors and seniors may be limited.

### 4.4. Future Research Directions

Future research should employ multi-wave longitudinal, intensive longitudinal, or intervention designs to clarify the temporal ordering and possible bidirectional association between EST and SWB. Combining device-recorded screen-use data, accelerometers, time diaries, and multi-source measures may improve measurement accuracy and reduce the influence of common method variance. Future studies should also distinguish among different entertainment activities and patterns of use, including active versus passive use and social versus nonsocial use, and directly measure immediate pleasure, recovery experiences, basic psychological need satisfaction, goal conflict, motivations for use, and displaced activities to test the proposed potential mechanisms. In addition, incorporating academic pressure, task demands, time-management difficulties, and psychological distress into the research model may help further clarify the contextual role of PTP. Finally, these findings should be replicated in samples from different regions, institutions, grade levels, and cultural backgrounds, and intervention studies are needed to evaluate their generalizability and practical significance.

## 5. Conclusions

The present study found a weak negative association between EST and SWB among college students, and this negative association weakened as PTP increased, indicating that PTP constitutes a contextual boundary condition of the association between EST and SWB. Therefore, future research should examine EST in conjunction with individuals’ levels of PTP rather than considering EST duration alone. The positive slope observed at extremely high levels of PTP involved only a small number of participants and should not be interpreted as indicating that increasing EST contributes to higher SWB.

## Figures and Tables

**Table 1:** Descriptive Statistics and Correlation Analysis (*N* = 1582).

Variable	*M*	*SD*	EST	SWB	PTP
EST	4.75	2.39	-		
SWB	21.73	14.22	−0.107 ***	-	
PTP	23.51	7.49	0.087 ***	−0.272 ***	-
MVPA	1.73	1.42	−0.076 **	0.056 *	−0.045

Note: *M* = mean, *SD* = standard deviation. EST = entertainment sedentary time; SWB = subjective well-being; PTP = perceived time poverty; MVPA = moderate-to-vigorous physical activity. EST and MVPA were expressed in hours per day. * *p* < 0.05, ** *p* < 0.01, *** *p* < 0.001.

**Table 2:** Multiple Linear Regression Analysis of the Association Between EST and SWB.

Predictor	*B*	*SE*	*β*	*t*	*p*	LLCI	ULCI
Constant	22.081	1.094		20.188	<0.001	19.935	24.226
Gender	0.565	0.724	0.020	0.780	0.436	−0.855	1.984
Sophomores	2.529	0.761	0.088	3.324	<0.001	1.037	4.022
Juniors	2.744	1.620	0.044	1.694	0.091	−0.434	5.922
Seniors	0.631	2.689	0.006	0.235	0.815	−4.643	5.905
MVPA	0.464	0.252	0.046	1.838	0.066	−0.031	0.958
EST	−0.642	0.150	−0.108	−4.279	<0.001	−0.936	−0.348

Note: *B* = unstandardized regression coefficient; *SE* = standard error; *β* = standardized regression coefficient; LLCI = lower limit of the 95% confidence interval; ULCI = upper limit of the 95% confidence interval. The confidence intervals refer to the unstandardized regression coefficients. Grade was dummy-coded, with freshmen serving as the reference group. Gender was coded as male = 0 and female = 1.

**Table 3:** Moderation Analysis of the Association Between EST and SWB.

Predictor	*B*	*SE*	*β*	*t*	*p*	LLCI	ULCI
Constant	19.269	0.828		23.285	<0.001	17.645	20.892
Gender	0.508	0.695	0.018	0.731	0.465	−0.855	1.872
Sophomores	2.425	0.730	0.085	3.324	<0.001	0.994	3.857
Juniors	2.670	1.554	0.043	1.718	0.086	−0.379	5.718
Seniors	2.581	2.584	0.024	0.999	0.318	−2.487	7.649
MVPA	0.283	0.243	0.028	1.167	0.244	−0.193	0.759
EST (X)	−0.558	0.145	−0.094	−3.853	<0.001	−0.842	−0.274
PTP (W)	−0.490	0.046	−0.258	−10.715	<0.001	−0.580	−0.401
X×W (Interaction)	0.085	0.019	0.108	4.485	<0.001	0.048	0.122

Note: *B* = unstandardized regression coefficient; *SE* = standard error; *β* = standardized regression coefficient. The 95% confidence intervals refer to the unstandardized regression coefficients. Grade was dummy coded, with freshmen as the reference group. EST and PTP were mean-centered before the interaction term was constructed. X represents EST, W represents PTP, and X × W represents the interaction between EST and PTP.

**Table 4:** Simple Slope Analyses of the Conditional Association Between EST and SWB.

Level of PTP	Value	*B*	*SE*	*t*	*p*	LLCI	ULCI
*M* − 1 *SD*	−7.488	−1.191	0.210	−5.663	<0.001	−1.604	−0.779
*M*	0.000	−0.558	0.145	−3.853	<0.001	−0.842	−0.274
*M* + 1 *SD*	7.488	0.075	0.194	0.386	0.700	−0.305	0.455

Note: PTP was mean-centered. Therefore, the centered PTP values of −7.488, 0.000, and 7.488 correspond to low (*M* − 1 *SD*), mean (*M*), and high (*M* + 1 *SD*) levels of PTP, respectively. The corresponding raw PTP scores were approximately 16.02, 23.51, and 31.00. B represents the conditional unstandardized regression coefficient for the association between EST and SWB. LLCI and ULCI represent the lower and upper limits of the 95% confidence interval, respectively.

## Data Availability

The data that support the findings of this study are not publicly available due to privacy and ethical restrictions but are available from the corresponding author upon reasonable request.
